# A Case of Occupational Asthma in a Plastic Injection Process Worker

**DOI:** 10.1186/2052-4374-25-25

**Published:** 2013-10-22

**Authors:** Jong Suk Lee, Hyun Seok Kwak, Byung Soon Choi, So Young Park

**Affiliations:** 1 Department of Occupational and Environmental Medicine, Eulji University Hospital, Daejeon, Korea; 2 Occupational Lung Diseases Institute, Korea Workers’ Compensation & Welfare Service, Ansan, Korea

**Keywords:** Occupational asthma, Plastic injection molding

## Abstract

**Objectives:**

We report a case of death due to asthma attack in a plastic injection process worker with a history of asthma.

**Methods:**

To assess task relevance, personal history including occupational history and medical records were reviewed. Samples of the substances utilized in the injection process were collected by visiting the patient’s workplace. The work environment with the actual process was reproduced in the laboratory, and the released substances were evaluated.

**Results:**

The medical records confirmed that the patient’s conventional asthma was in remission. The analysis of the resins discharged from the injection process simulation revealed styrene, which causes occupational asthma, and benzenepropanoic acid, 3,5-*bis*(1,1-dimethylethyl)-4-hydroxy-, and octadecyl ester. Even though it was not the case in the present study, various harmful substances capable of inducing asthma such as formaldehyde, acrolein, and acetic acid are released during resin processing.

**Conclusion:**

A worker was likely to occur occupational asthma as a result of the exposure to the harmful substances generated during the plastic injection process.

## Background

Work-related asthma is classified as either occupational asthma, which occurs as a result of exposure to certain causative substances or stimulants present in the work environment, or work-aggravated asthma, which is conventional asthma that worsens because of the work environment. Work-related asthma is the most common occupational lung disease in industrialized countries [1-3] and is the second most common occupational lung disease in Korea after pneumoconiosis [4].

A study [5] on occupational asthma conducted on a sample population of 15,637 individuals in 12 industrialized countries including Spain and Sweden specifies farmers, painters, plastic and rubber production workers, and janitors among others as high-risk occupational groups for occupational asthma compared to administrative and office workers. In particular, plastic manufacturing incurs the third largest odds ratio (OR) of asthma occurrence (OR: 2.2, 95% confidence interval [CI]: 0.59–8.29), behind farmers (OR: 2.62, 95% CI: 1.29–5.35) and painters (OR: 2.34, 95% CI: 1.04–5.28). According to the Statistics by Group of Industry (5 or more workers) in 2010 provided by Statistics Korea [6], there were 9,058 plastic and rubber product manufacturers for a total of 210,000 employees. In Korea, the annual incidence of work-related asthma in plastic and rubber product manufacturing is 9.29 cases per million people; in addition, people working in furniture, chemical, vehicle, and food and beverage manufacturing are at high risk of asthma [7]. However, there are no reports of cases of occupational asthma related to the plastic molding process.

Here, we experienced a fatal case that was presumably due to the aggravation of new-onset sensitization-induced asthma in a plastic injection worker with a history of asthma. This case is of interest because plastic processing is a large manufacturing sector in Korea. Despite this, the risks of work environments inducing asthma are not well understood. Therefore, more attention in occupational and environmental medicine is required in this industry.

## Case presentation

### Subject

A 47-year-old man.

#### Onset and progress of bronchial asthma

The patient had a chief complaint of cough that persisted for one month and presented to the Respiratory Clinic of the University Hospital in July 1998. At the time of admission, wheezing was not auscultated in the chest and chest radiographs were normal. On spirometry, forced vital capacity (FVC) was 3.84 L (90.4% predicted), forced expiratory volume in 1 second (FEV_1_) was 2.76 L (71.9% predicted), and the FEV_1_/FVC ratio was 71.9. A methacholine challenge test revealed mild bronchial hyperreactivity (provocative concentration producing a 20% decrease in FEV_1_ (PC20) to 8.0 mg/mL). Asthma was diagnosed on the basis of the asthma symptoms and nonspecific bronchial hyper reactivity of the patient.

Despite the diagnosis of asthma, he had been living without any symptoms, had not been receiving treatments, and he gained employment at an automotive bumper and vehicle control display mold manufacturer in March 2004 and worked on grinding. He started working on a plastic injection process simulation in May 2006. His cough and breathing difficulties became aggravated starting in 2010, approximately 4 years after starting work in injection molding. In December 2011, he presented to the emergency department of a local hospital at around 23:00 hours, prompted by breathing difficulties while resting after work. An arterial blood gas analysis on admission revealed hypoxia with an arterial oxygen partial pressure of 50.2 mmHg and oxygen saturation of 86%. Chest radiographs did not indicate abnormalities. The peripheral blood examination manifested normal leukocyte counts (10,000/μL), whereas the eosinophil percentage was elevated (7.5%; reference range, 0–7%). Therefore, he was considered to have had an asthma attack and was subsequently hospitalized, where he received oxygen and bronchodilator treatments. Spirometry conducted during hospitalization revealed respiratory obstruction with a FVC of 1.87 L (57% of predicted), FEV_1_ of 1.23 L (46% of predicted), and a FEV_1_/FVC ratio of 65.8. The patient presented to the emergency department again because of an asthma attack in March 2012.

In June 2012, the patient finished overtime work at 21:10 hours and visited a massage parlor 5 minutes driving distance from the company at around 22:30 hours. After being seated and waiting 20 minutes for his appointment, he suddenly had breathing difficulties. He was evacuated to the local hospital but ultimately died. At the hospital he was transferred to, he exhibited resistance during ambu bagging. In addition, expiratory wheezing was heard from both lungs during physical examination, which led to the diagnosis of acute asthma exacerbation. In accordance with the request of the bereaved, the National Forensic Service conducted an autopsy and concluded the death was caused by an acute asthma attack.

The health insurance reimbursement history from 2002 confirmed that the patient received 2 or 3 treatments per year at the clinic that prescribed his inhaler. He underwent 11 treatments in 2010, which was 4 years after starting work with the injection process; the number notably increased to 29 times in 2011.

His colleagues knew he had asthma but did not notice any abnormalities during regular working hours. They mentioned that he coughed frequently and felt out of breath after dinner on the day of his death because of the amount of work he had performed.

#### Personal and medical history

Nothing specific was found in his medical history except for the aforementioned bronchial asthma. Furthermore, he smoked one pack of cigarettes daily for 24 years. A health check-up performed in 2011 did not reveal any abnormal findings except for obesity.

#### Workplace environment and job description

The patient started working in an automotive bumper and vehicle control display mold manufacturer in March 2004 at the age of 39 years and died due to the asthma in June 2012 at 47 years of age. His working procedure was as follows: die design→machining→grinding→injection process simulation and inspection. The patient ground the processed mold at the time of admission and was working on product injection process simulation and inspection of the finished mold from May 2006.

**Figure 1:**
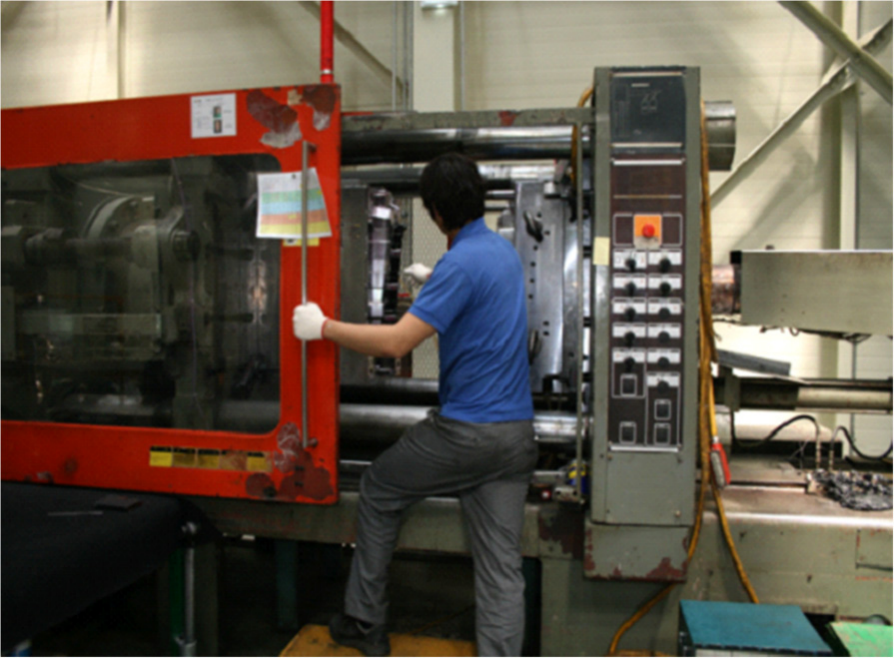
Operation of the injection molding machine and ejected mold-releasing agents.

**Figure 2:**
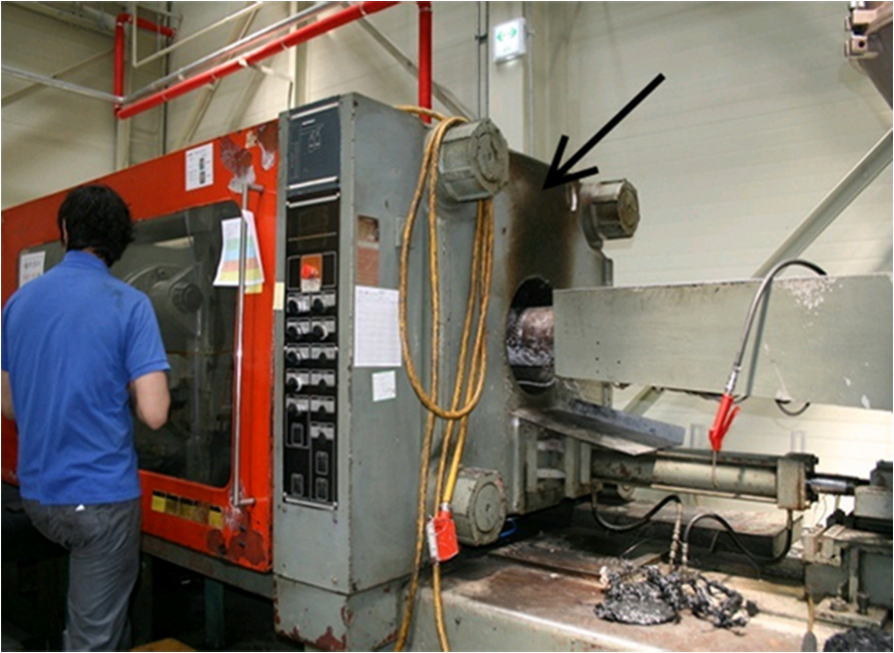
**Injection port of the plastic resins (pouring resins into the hopper of the molding machine).** The arrow indicates discoloration caused by hume leakage when injection processing or changing the raw material.

Injection process simulation is a working process in which injection is performed to test the correct functioning of a new mold. In the injection process simulation, mold-releasing agents are sprayed onto the prepared mold. When the injection molding machine is closed, acrylonitrile-butadiene-styrene (ABS), polyethylene (PE), polypropylene (PP), nylon, acetal, and polybutylene terephthalate (PBT) resins in pellet form are automatically injected to the die and heated. After around 2–3 minutes, the products are pulled out from the injection molding machine and inspected (Figure 1). The average daily workload of this work process involves injection process simulations of 3–7 dyes; the types of resin and duration of handling widely vary depending on working circumstances. During the process of opening the injection molding machine and removing the products, workers can be exposed to fumes released from the melted resins. The risk of exposure to a large amount of fumes is especially high while the residual resin left in the injector is removed to change the dye (Figure 2).

The injection temperature differs with respect to the resin used as follows: 240–260°C, 230–250°C, 280°C, 220°C, and 250–300°C for ABS, polyethylene and polypropylene, nylon, acetal, and PBT resins, respectively.

Lubricating rust inhibitors (i.e., WD-40), mold-releasing agents (i.e., KF96 spray), slideway oil (i.e., Mobil Vactra Oil No.4), and hydraulic oil (i.e., Mobil DTE24) are utilized in the injection process simulation. Slideway and hydraulic oil are intermittently added to the machine, and lubricating rust inhibitors and mold-releasing agents are mainly handled by the workers. The major components of the 2 products from material safety data sheet (MSDS) are WD-40 comprises hydrotreated light distillate (concentration: 30–40%), solvent-dewaxed heavy paraffinic distillate (concentration: 6–12%), isobutane (concentration: 8–12%), *n*-butane, (28~32%), and trade-secret material (concentration <12%). The KF96 spray comprises dimethylpolysiloxane (concentration: 5%) and liquefied petroleum gas, (concentration: 95%). None of these components are asthma triggers.

There was no local ventilation system in the vicinity of the injection molding machine, and the molds were produced in a separate factory building not adjacent to the workplace of the deceased the patient. In the workplace where he used to work with injection molding processes, only noise in the working environment had been assessed at that time.

#### Analysis of the pyrolyzed resins

Because different resins are used for different durations depending on the work situation, it was inappropriate to assess all resins used by the deceased the patient through working environment measurements. Therefore, the resins utilized in the workplace were collected, and the injection process was reproduced by heating the resins at the actual injection temperature to assess the resultant substances. With exception of PBT resins, which we were unable to obtain from the site, 5 resins in pellet form utilized in the injection process, namely ABS, polyethylene, polypropylene, nylon, and acetal resins, were heated at their actual injection temperatures by a pyrolyzer (Model EGA/PY-3030D, Frontier Lab Ltd.). The products were qualitatively analyzed by a gas chromatography mass analyzer (Agilent Technologies 7890A GC, 5975C MSD; Table 1). Styrene, which is a known asthma-inducing substance, benzenepropanoic acid, 3,5-*bis*(1,1-dimethylethyl)-4-hydroxy-, and octadecyl ester were detected from the ABS resin.

**Table 1:** Analysis of the pyrolyzed products of the heated resins (pyrolyzed products from heated resins)

**Materials**	**Temp**^**†**^**(°C)**	**Area(%)**	**Library/ID**	**CAS No.**^**‡**^	Quality^*^(%)
ABS^§^	240~260	2.02	Styrene	100-42-5	97
		1.66~1.72	2-PROPENOIC ACID, DODECYL ESTER	2156-97-0	90~91
		0.52	Benzene,1,1′-(1,2-cyclobutanediyl)bis-,cis-	7694-30-6	90
		0.4	OCTADECANE	593-45-3	98
		0.59~0.78	EICOSANE	112-95-8	96~99
		2.35~5.44	3-[1-(4-Cyano-1,2,3,4-tetrahydronaphthyl)]propanenitrile	57964-40-6	98
		2.95	OCTADEC-9-ENOIC ACID	112-80-1	99
		0.51	DOCOSANE	629-97-0	99
		7.76	Benzenepropanoic acid, 3,5-bis(1,1-dimethylethyl)-4-hydroxy-, octadecyl ester	2082-79-3	90
		5.2	2-Propenoic acid pentadecyl ester	43080-23-5	91
		4.86~12.02	2-[1-(4-Cyano-1,2,3,4-tetrahydronaphthyl)]propanenitrile	57964-39-3	91~95
PE^∥^	230~250	0.55	1-Octadecene	112-88-9	96
		0.16	EICOSANE	112-95-8	94
		0.74	HENICOSYL FORMATE	77899-03-7	91
		0.19	Heneicosane	629-94-7	90
		0.16	HEPTACOSANE	593-49-7	91
PP^¶^	230~250	6.03	Hexadecane	544-76-3	90
		2.94	Octacosane	630-02-4	90
		5.17~5.53	TRICOSANE	638-67-5	91
		2.44	TRIACONTANE	638-68-6	90
		0.19	Heneicosane	629-94-7	90
		0.16	HEPTACOSANE	593-49-7	91
Nylon	280	100	Caprolactam	105-60-2	93

In the acetal resins, components with a quality higher than 90% were not detected at 220°C.

^*^Quality means matching ratio to database.

^†^Temp: temperature, ^‡^CAS No.: chemical abstractions service registry number, ^§^ABS: acrylonitrile-butadiene-styrene, ^∥^PE: polyethylene, ^¶^PP: polypropylene.

## Conclusion

The present case of fatal asthma attack was due to acute exacerbation of work-related asthma that developed in a plastic injection process worker with a history of asthma in remission. The deceased patient led a normal life without manifestations of asthma symptoms or any treatments. However, he began to exhibit typical late-stage asthma symptoms 4 years after working with injection process simulation. Unaware of the occupational cause of those symptoms, he continued to work and finally succumbed to an asthma attack.

Occupational asthma is classified as sensitizer- or irritant-induced asthma depending on the presence or absence of sensitization and a latency period. Sensitizer-induced asthma occurs as a result of immunologic sensitization and is preceded by a latency period after exposure to asthma-triggering substances in the workplace [8]. The latency period between exposure to the causative substances and manifestations of symptoms may range from several weeks to years depending on the phase of the causative substance [9]. Irritant-induced asthma occurs without any latency period after inhaling a high concentration of an irritant. Referring to irritant-induced asthma, Brooks et al. [10] defined reactive airways dysfunction syndrome as (i) the development of respiratory symptoms such as coughing, wheezing, and dyspnea (i.e., shortness of breath) within 24 hours of a high-dose exposure to irritants that persist over 3 months; (ii) airflow obstruction in pulmonary function testing; and (iii) positive response to a methacholine challenge test. Burge et al. [11] reported cases of irritant-induced asthma with a latency period after repeated low-level exposures to irritants. Although the pathophysiological mechanism of irritant-induced asthma is unclear, it is assumed to arise from damage to the bronchial epithelium after inhaling high concentrations of irritants; this leads to the loss of the innate function of epithelial host defense, weakening of the relaxing mechanism of epithelium, and neurogenic inflammation [12]. Asthma exacerbated by occupational environment involves the aggravation of symptoms in workers with preexisting asthma or asthmatic symptoms due to nonspecific causes such as irritants, allergens, fungi, cold workplace temperature, emotional stress, and physical exertion due to overwork [13]. While this type of asthma exhibits symptoms similar to those of occupational asthma, it differs in that it presents with more intense airflow obstruction in pulmonary function testing and a higher rate of healthcare resource utilization as an outpatient or emergency department patient. Workers with work-exacerbated asthma are not sensitive to irritants in workplace; in many cases, patients exhibit no change in airway inflammation [14].

The plastic production process involves the use a variety of additives such as resins, plasticizers, and stabilizers. During the process of producing resins, which are the raw materials for plastic, and plastic products using the prepared resins, asthma can be triggered by the pyrolytic decomposition products discharged from resins and additives including those generated during their heating. An epidemiological survey conducted by the National Institute for Occupational Safety and Health described the case of a worker in his 20s who developed asthma after working for 2 years at an unsaturated polyester synthetic resin manufacturer. His work involved inserting fuel into a reaction vessel. The fuel comprised liquid-phase diethylene glycol and dipropylene glycol as well as pulverized phthalic anhydride [15] and anhydrous maleic anhydride [16]; these 2 powdered fuels to which he was exposed are known asthma-triggering substances. Asthma symptoms began to manifest after 1 year of exposure. This case was categorized as occupational asthma directly associated with the work environment [17]. There is another case report of a 47-year-old worker who died of a suspected asthma attack involving bronchial asthma and chronic obstructive pulmonary disease (COPD), which he concurrently developed after working for 10 years at a factory manufacturing cosmetic containers made of synthetic resins. The deceased patient performed machinery inspection, pigment mixing, and container packaging in an enclosed space. Working environment measurements detected low-level exposure to formaldehyde, acetaldehyde, and diphenylamine, thus confirming a high probability that the asthma was induced by these substances [18]. Park et al. [19] report a case of reactive airways dysfunction syndrome (RADS) caused by high-level exposure to harmful substances at a factory manufacturing semi-finished synthetic resin products. The worker was exposed to high-concentrations of aerosols and gases during the operation of an additive mixer in the mixing process after injecting polyvinyl chloride (PVC) as a raw material; DEHP (di-(2-ethylhexyl) phthalate, dioctyl phthalate (DOP), epoxidized soybean oil, and dibutyltin maleate as plasticizers and stabilizers; and KCZ-05 as an additive. The worker developed acute symptoms of dyspnea and coughing immediately after exposure. His physical examination confirmed wheezing and reduced respiratory sound; pulmonary function testing confirmed airflow obstruction, showing reduced values of the forced expiratory volume in 1 second and liters per second. Thus, it was assumed that the exposure to the pyrolytic decomposition products as well as the raw materials and additives themselves were the causative agents of RADS. In addition, a wide variety of other substances is known to trigger asthma, such as azodicarbonamide, which is used as a blowing agent for plastic and rubber [20,21].

ABS, polyethylene, polypropylene, nylon, acetal, and PBT resins are utilized as raw materials for a wide range of plastic products from industrial materials to consumer merchandise. All of these resins are thermoplastics that melt and become highly viscous liquids when heated and are high-molecular-weight substances that solidify when cooled. Workers can be exposed to various substances during the thermal process for molding.

The analysis of the pyrolysis products in this study revealed the presence of styrene, a known asthma-inducing substance; benzenepropanoic acid; 3,5-*bis*(1,1-dimethylethyl)-4-hydroxy-; and octadecyl ester in the ABS resin.

Even though there are no instances in which benzenepropanoic acid, 3,5-*bis*(1,1-dimethylethyl)-4-hydroxy-, oroctadecyl ester added as stabilizer were reported to induce asthma, the Hazardous Substance Data Bank [22] classifies these as substances that should be treated with caution because they can cause skin and respiratory system irritation, asthma, and/or allergic dermatitis.

Styrene, a volatile organic compound, is commonly utilized in plastic manufacturing processes, furniture, and automobile repair factories using paint that includes polyester resins. It is mainly known to induce skin and mucous membrane irritation and nervous system toxicity. It is also known as an asthma-inducing substance. Moscato et al. [23] reported the cases of a 31-year-old man with asthma that occurred 2 months after working in a polystyrene production factory and a 45-year-old woman with asthma that occurred 5 years after she started working in a plastic button factory using polyester resins. Both cases presented with early asthmatic responses for the specific inhalation test against styrene. Hayes et al. [24] published a case of asthma in a 30-year-old air force aircraft mechanic who exhibited dual responses on the specific inhalation test against styrene. During the specific inhalation test, the atmospheric styrene level was 12 ppm (reference level, 100 ppm) and the asthmatic response manifested even at the level that mandelic acid (a metabolite in blood and urine) was not detected. Fernandez-Nieto et al. [25] published a case of a 31-year-old automobile mechanic who used paint that included polyester resins; the patient presented with late asthmatic responses on the specific inhalation test against styrene with elevated eosinophils and basophils, indicating bronchial inflammation in sputum induction. In Korea, a case of a man in his 50s who was working with putty as part of a railroad vehicle repair process was reported; the man had asthma caused by direct styrene exposure from the putty and toluene diisocyanate (TDI) during spray painting of the local workplace [26]. When measuring the patient’s work environment at that time, 19-ppm styrene was detected in the putty process. Moreover, the specific inhalation test results for styrene and TDI were positive. Therefore, the man’s condition was diagnosed as occupational asthma caused by these substances. A worker in his 30s, whose job was curing tires by inserting green tires into a tire mold and applying pressure and heat, developed asthma symptoms after 4 years of work. Rubber fumes ranging from 0.18–0.80 mg/m^3^ and a maximum styrene level of 0.19 ppm were detected. His average peak expiratory flow decreased during working hours (361.7 L/min) compared with that during rest days (417.1 L/min). In addition, a 15% decrease during working hours compared to resting hours within the same day was an obvious indicator of the occupational relevancy in the development of asthma symptoms [27].

Various pyrolyzed products can be formed during the plastic manufacturing process when heated to the recommended temperature or higher; polypropylene can generate formaldehyde, acrolein, acetone, polyethylene can produce alkanes including butane and alkenes, and acetal resins can create formaldehyde [28].

Malo et al. [29] reported a case of asthma occurring in a worker at a bag factory using polypropylene. The affected worker was in charge of operating a machine that makes polypropylene pellet fabrics by heating them at 250°C. The specific inhalation test performed by exposure to the thermal process of the polypropylene revealed late asthmatic responses. Various substances such as aliphatic hydrocarbons (e.g., ethylene and butane), aldehydes (e.g., formaldehyde), and ketones can form during the heat treatment of polypropylene. Therefore, the specific inhalation test for formaldehyde, which is a well-known asthma-inducing substance, was performed. However, there was no significant change in FEV_1_. Consequently, this suggests that heated polypropylene itself and not formaldehyde can cause asthma.

Skerfving et al. [30] reported that bronchospasm or “meat wrappers’ asthma” can be induced by polyvinyl chloride heated by a crimp film machine in the meat packaging processor hume occurring in the price tag. Polyethylene films were cut by a wire heated to 200°C, and the crimp film machine, which compresses films in a tunnel oven at 220°C, was introduced to the workplace. The worker experienced breathing difficulties, wheezing, and coughing every night the crimp film machine was operated. The symptoms were reduced after ventilation was installing and disappeared after the work transition. The authors suggest that hume produced as a result of the thermal treatments of polyethylene could induce asthma. Gannon et al. [31] reported a case of asthma in a worker that developed after shrink wrapping using polyethylene sheets heated to 166°C. The worker exhibited dual responses on the specific inhalation test when exposed to polyethylene heated to 76°C. Polyethylene, and acrolein and acetic acid can generate formaldehyde when heated at 300°C and 400°C or higher, respectively. However, in this case, the asthmatic response manifested when the polyethylene was heated at low temperature. Thus, the polyethylene itself was able to cause asthma and not the substances produced when it was heated.

Cartier et al. [32] published a case of an asthma and alveolitis-type reaction due to polyester paint. Power paint comprising polyester (14%) including epoxyresins (86%), polyethylene terephthalate, andpolybutylene terephthalate was sprayed onto metallic boards and heated in an oven at 200°C. The worker worked on removing the painted metallic boards from the oven. However, the worker did not exhibit any responses on specific inhalation tests using epoxy resins and trimellitic anhydride (TMA) but manifested systemic responses including high fever, leukocytosis, and a decrease in diffusing capacity along with positive responses against polyester powders.

The cases described above confirm that asthma can be triggered by exposure to chemical intermediates produced during the plastic resin handling process, such as phthalic anhydride and maleic anhydride, as well as additives such as DEHP, epoxidized soybean oil, dibutyltin maleate, KCZ-05, and azodicarbonamide. In addition, asthma can be triggered by both resins themselves and their decomposition products during plastic resin or rubber handling processes as well as by paints containing plastic resin. In these cases, asthma symptoms began to manifest between 2 weeks and 10 years after exposure to causative substances. The severity varied, demonstrating early to late asthmatic responses as well as dual responses in the bronchial challenge test.

The patient in the present report obtained an asthma diagnosis in 1998, but the methacholine challenge test showed extremely mild bronchial hyperreactivity (PC_20_) at that time (8.0 mg/mL). His asthma was subsequently under control with bronchodilators administered 2 or 3 times per year. He began working at an automotive bumper and vehicle control display mold manufacturer in March 2004 and worked on injection process simulation in May 2006. From 2010, approximately 4 years after starting the injection process, he attended the clinic more frequently and even attended the emergency department because of acute asthma attacks. The patient eventually died because of a sudden asthma attack that occurred around 23:00 hours in June 2012 after working overtime until 21:00.

Because the patient had asthma prior to joining the company, his conventional asthma was possibly aggravated by his work. However, the asthma symptoms of the patient remained unchanged for the first 6 years after the admission (i.e., 4 years after starting the injection process work), and levels of the gas and hume generated by the injection process were not considerably high. In addition, his aggravated asthma symptoms after 2010 manifested mainly at night after work. His colleagues also mentioned that he did not have specific symptoms during the daytime, although he coughed frequently and felt out of breath at night on the day he worked overtime. Therefore, the patient is considered to have a late asthmatic response in which symptoms presented several hours after exposure to the causative factors. If the asthma of the patient is assumed to be work-aggravated asthma exacerbated by nonspecific stimulants such as highly concentrated dust, gas, or hume in the workplace, the aggravated symptoms should have manifested prior to 2010 and developed during working hours rather than at home after work. Therefore, the asthma of the patient cannot be work-aggravated asthma.

The plastic injection process facilitates exposure to various substances generated by the pyrolysis of resins melting at high temperature. The substances generated when ABS, polyethylene, polypropylene, nylon, acetal, and PBT resins were heated at the actual injection temperature were analyzed; styrene, benzenepropanoic acid, 3,5-*bis*(1,1-dimethylethyl)-4-hydroxy-, and octadecyl ester were detected. Among the substances formed as a result of heating the aforementioned resins, formaldehyde [21], acrolein [33], and acetic acid [34] are reported to be causative substances of asthma. Heated polypropylene and polyethylene can also induce asthma. Therefore, although the causative substances are unknown, the patient appears to have died because of aggravation of new occupational asthma that occurred as a result of exposure to various asthma-inducing substances generated by the plastic injection process.

According to the deaths by cause data in 2011 in Korea (Statistics Korea) [35], deaths due to asthma are relatively rare at 3 per 100,000 people. However, workers with occupational asthma are a high-risk group for near-fatal asthma and present to the emergency department with ORs of 3.5 (95% CI, 1.5–8.0) and 3.0 (95% CI, 1.2–7.7), respectively, compared with patients with non-occupational asthma [36]. In the case of the patient, the number of times he attended the clinic because of asthma increased markedly since 2010. Starting from a year before his death, he visited the emergency department twice because of breathing difficulties and was hospitalized once. However, no particular actions were taken in his workplace. Because his symptoms were late asthmatic responses manifesting mainly after the work and not during work and because he had a history of asthma and was unaware of exposure to asthma-causing substances in the plastic injection process, he would not have realized his asthma was associated with his work. Therefore, harmful substances that may be generated during the plastic manufacturing and their health effects need to be clearly understood and active education should be performed accordingly to prevent deaths like the present case.

In the present study, we were unable to perform a specific inhalation test useful for classifying the patient’s occupational and work-aggravated asthma, investigate changes in peak expiratory flow rates during work, monitor nonspecific bronchial hyperreactivity (PC_20_) of methacholine, or confirm eosinophils in the sputum examination because he was already dead at the time of the study [14,37]. Although the working process that led to the release of asthma-triggering substances was objectively reproduced by heating the resins handled by the deceased the patient to the actual temperature in the plastic injection molding process and analyzing the discharged substances, the present study is still limited by a lack of proof that these substances actually triggered his asthma symptoms. Nevertheless, the present study is significant in that it verifies that asthma can occur as a result of sensitization to low concentrations of harmful substances. In addition, detailed investigations of medical records and occupational history revealed that exposure to causative substances in the workplace can trigger the incidence of asthma. These results can serve as a basis for determining the occupational relevancy of asthma cases.

To date, no cases of asthma occurring during plastic processing have been reported in Korea. However, greater attention and additional studies focusing on the plastic industry, which has a high potential risk of asthma occurrence, are required.

## Consent

Written informed consent was obtained from the patient’s family/colleague for the publication of this report and any accompanying images.

## Competing interests

The authors declare that they have no competing interests.

## Authors’ contributions

JSL, SYP, BSC conceived and designed the study. HSK performed the analysis and interpretation of data. LJS, PSY were involved in writing the manuscript. All authors read and approved the final manuscript.
